# Novel *FOXL2* mutations cause blepharophimosis‐ptosis‐epicanthus inversus syndrome with premature ovarian insufficiency

**DOI:** 10.1002/mgg3.366

**Published:** 2018-01-29

**Authors:** Xiao‐Wen Yang, Wen‐Bin He, Fei Gong, Wen Li, Xiu‐Rong Li, Chang‐Gao Zhong, Guang‐Xiu Lu, Ge Lin, Juan Du, Yue‐Qiu Tan

**Affiliations:** ^1^ Institute of Reproductive and Stem Cell Engineering Central South University Changsha Hunan China; ^2^ Reproductive and Genetic Hospital of Citic‐Xiangya Changsha Hunan China

**Keywords:** blepharophimosis‐ptosis‐epicanthus inversus syndrome, FOXL2, novel mutation, premature ovarian insufficiency

## Abstract

**Background:**

Blepharophimosis‐ptosis‐epicanthus inversus syndrome (BPES) is a malformation of the eyelids. Forkhead Box L2 (*FOXL2*) is the only gene known to be associated with BPES.

**Methods:**

We identified two Han Chinese BPES families with premature ovarian insufficiency (POI). Sanger sequencing and in vitro functional analysis were performed to identify the genetic cause.

**Results:**

Sanger sequencing identified two novel mutations (c.462_468del, c.988_989insG) in *FOXL2,* one in each family. The in vitro functional analysis confirmed that both novel mutations were associated with impaired transactivation of downstream genes. Specifically, the single‐base insertion, c.988_989insG, led to subcellular mislocalization and aggregation of the encoded protein, which validated the hypothesis that the two novel *FOXL2* mutations are deleterious and associated with POI in the two BPES families.

**Conclusion:**

The novel mutations identified in the present study will enhance the present knowledge of the mutation spectrum of *FOXL2*. The in vitro experiments provide further insights into the molecular mechanism by which the two new variants mediate disease pathogenesis and may contribute to elucidating the genotype‐phenotype correlation between the two novel *FOXL2* mutations and POI.

## 1 INTRODUCTION

Blepharophimosis‐ptosis‐epicanthus inversus syndrome (BPES, OMIM 110100) is an autosomal dominant condition characterized by eyelid malformations, involving ptosis, a narrowed horizontal palpebral aperture, epicanthus inversus, and telecanthus. The incidence of BPES is approximately 1 per 50,000 in the general population (Oley & Baraitser, 1988). BPES may be categorized into two clinical subsets based on the additional presence or absence of premature ovarian insufficiency (POI). BPES type I manifests with palpebral malformations associated with POI, whereas patients with BPES type II exhibit isolated eyelid malformations (Zlotogora, Sagi, & Cohen, 1983). Both types of BPES are caused by mutations in the Forkhead Box L2 gene (*FOXL2*, OMIM 605597).

*FOXL2*, which encodes a forkhead transcription factor, is the only gene known to be associated with BPES. Apart from the mesenchyme of developing eyelids, *FOXL2* is highly expressed in granulosa cells of the ovary and gonadotropic cells of the anterior pituitary (Batista, Vaiman, Dausset, Fellous, & Veitia, 2007; Cocquet et al., 2003; Ellsworth et al., 2006). The pattern of its expression suggests that FOXL2 might play a pivotal role in regulating the development of the ovaries and maintaining the female gonads in vertebrate species (Cocquet et al., 2003; Leung, Fuller, & Chu, 2016). The vast majority of variations that lead to truncated FOXL2 are generally related to BPES type I, while the variations that result in full‐length proteins are often associated with preserved ovarian function and BPES type II (Crisponi et al., 2001). To date, numerous germline mutations in *FOXL2* have been identified to be responsible for BPES with POI (Kaur, Hussain, Naik, Murthy, & Honavar, 2011), whereas the genotype‐phenotype correlation between FOXL2 mutation and the type of BPES developed has not yet been fully delineated.

In this study, we identified two novel mutations of *FOXL2* in two Han Chinese families with BPES type I by screening *FOXL2*. The aim of the present work was to comparatively evaluate the effect of the novel mutations on FOXL2 bioactivity via in vitro functional analysis and delineate the correlation between the two novel FOXL2 mutations and the types of BPES. The present insights into these novel mutations might contribute to improving diagnostics, genetic counselling, as well as fertility guidance in clinical settings. The results of the endocrine examination, FOXL2 sequencing, and in vitro functional analysis contributed to elucidating the genotype‐phenotype correlation between the two novel *FOXL2* mutations and POI.

## 2 MATERIALS AND METHODS

### 2.1 Patients

**Figure 1:**
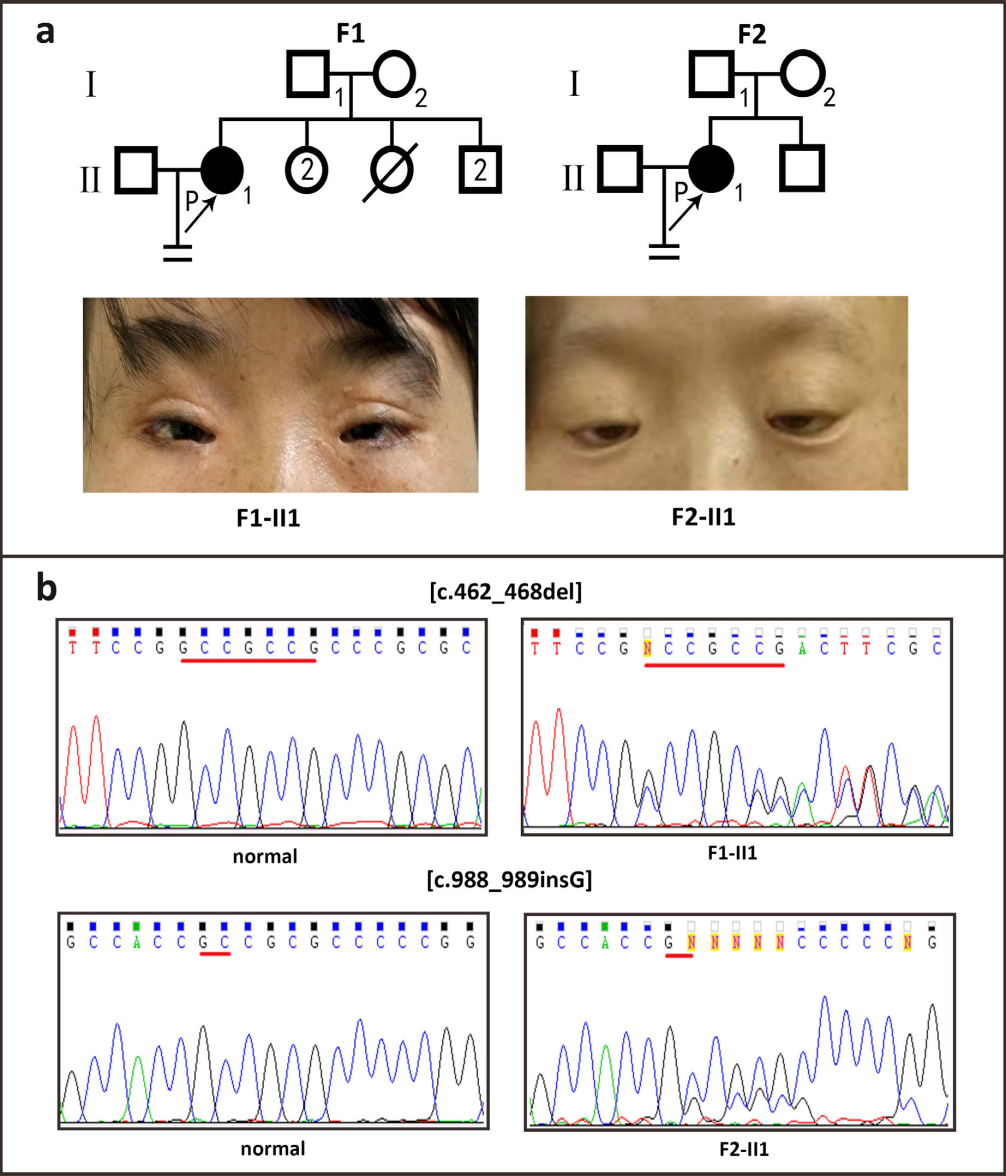
Pedigree and Sanger sequencing analysis of the probands from the two Han Chinese families. Panel a: Pedigree and eyelid photographs of the probands from two families in this study before or after surgery. Affected members are indicated by filled symbols; unaffected relatives are indicated by open symbols. The number of siblings is indicated in the symbol. The arrow indicates the proband. Numbers are allotted to family members whose DNA samples were used in this study. Panel b: Sanger sequencing analysis of the two families in this study.

Two probands from two families were admitted to the Reproductive and Genetic Hospital of CITIC‐Xiangya due to eyelid malformations and primary infertility (Figure 1a). The probands received detailed ophthalmic examinations by ophthalmologists and were diagnosed with BPES based on the following criteria: blepharophimosis, ptosis, epicanthus inversus, and telecanthus. Photographs of the probands’ eyelids were taken before or after surgery for assessment of BPES‐related features (Figure 1a).

Premature ovarian insufficiency, which is defined as the depletion or loss of normal ovarian function, is characterized by the acquisition of hypergonadotropic hypogonadism in women before the age of 40 years (Caburet et al., 2014; The ESHRE Guideline Group on POI et al., 2016). All affected female participants in this study presented with elevated serum gonadotrophin concentrations and primary infertility and had received the diagnosis of POI in the locality according to the latest criteria (The ESHRE Guideline Group on POI et al., 2016). The probands of family F1 and F2 experienced irregular menstrual cycles; the proband from family F2 presented with a decline in antral follicle count, indicating decreased ovarian reserve. Since they received traditional Chinese medicine treatments after their irregular cycles and we could not obtain the hormonal characteristics and clinical features of the probands before treatment, the information on these features after the treatment is provided in Table 1.

**Table 1:** Hormonal characteristics and clinical features of two probands after the reatment of trational Chinese medicine

Patient	Age/years	LH (mIU/ml)	FSH (mIU/ml)	Prolactin (ng/ml)	Estradiol (pg/ml)	Progesterone (ng/ml)	Testosterone (ng/ml)	Follicle numbers	Clinical information
F1‐II1	34	6.58	17.49↑	19.55	40	0.15	0.85	Left: 5, Right: 10	BPES; Menarche 16 years; irregular cycles
F2‐II1	30	10.33	17.84↑	7.16	42	0.2	0.59	Left: 2, Right: 3	BPES; Menarche 15 years; irregular cycles

Ref. value of female follicle period: LH: 1.80–11.78 mIU/ml, FSH: 3.03–8.08 mIU/ml, Prolactin: 5.18–26.53 ng/ml, Estradiol: 21–251 pg/ml, Progesterone: <0.1–0.3 ng/ml, Testosterone: 0.38–1.97 ng/ml.

All affected women were found to have a normal 46, XX karyotype, and FMR1 CGG repeats in the normal polymorphic range. No associated endocrinopathies or autoimmune disorders were found. Informed consent was obtained from all participants in this study, which was approved by the ethics committee of the Reproductive and Genetic Hospital of CITIC‐Xiangya.

### 2.2 Sequencing and variant analysis

Genomic DNA was extracted from whole‐blood leucocytes of patients using a QIAamp Blood DNA Mini kit (Qiagen, Hilden, Germany) according to the manufacturer's instructions. The entire coding region and intron‐exon boundaries of *FOXL2* were amplified from genomic DNA using polymerase chain reaction (PCR) as previously described (Bell, Murday, Patton, & Jeffery, 2001) and directly sequenced using the Applied Biosystems 3130 genetic analyser (ABI, USA). Subsequently, bioinformatics analysis of all identified mutations was performed using MutationTaster (http://www.mutationtaster.org/) and Combined Annotation Dependent Depletion (CADD, http://cadd.gs.washington.edu/).

### 2.3 Plasmid constructs

Since *FOXL2* is a single‐exon gene, its full‐length open reading frame was amplified from the genomic DNA of patients using primers incorporating restriction enzyme sites (EcoRI‐KpnI). The amplified gDNA products were purified, digested, and then cloned into the digested plasmids of phosphorylated enhanced green fluorescent protein (EGFP)‐N1 (Clontech, CA, USA), leading to the production of fusion proteins with the EGFP on the C terminus of FOXL2 (the former codon of termination codon in the wild‐type FOXL2 or predicted termination codon in mutant FOXL2 followed by the open reading frame of EGFP, which were designated as FOXL2‐WT‐EGFP, FOXL2‐Pro156Argfs‐EGFP, and FOXL2‐ Ala330Glyfs‐EGFP, respectively). All expression constructs were sequenced to confirm the presence of the desired mutations and exclude PCR‐induced mutations.

### 2.4 Cell culture and transient transfection

CHO cells were seeded at a density of 2.5 × 10^5^ cells in a 12‐well plate and allowed to grow to 70%–80% confluence prior to transfection under standard conditions in complete media (DMEM/F12 [Gibco, USA] and 10% FBS at 37°C with 5% CO_2_). CHO cells were transiently transfected with expression vectors containing wild‐type or mutated proteins, using Lipofectamine^®^ 2000 (Thermo Fisher Scientific, Carlsbad, CA, USA) according to the manufacturer's instructions. Cells transfected with an empty vector (without *FOXL2*) served as the negative control. The transfected cells were incubated for 36 hr prior to their preparation for immunofluorescence and RNA extraction.

### 2.5 Immunofluorescence and confocal microscopy

Immunofluorescence was performed as previously described (Caburet et al., 2001) with transfected CHO cells grown on coverslips. Fluorescent images were captured by confocal microscopy (Olympus FV1000, Tokyo, Japan).

### 2.6 Quantitative real‐time PCR

It has been reported that FOXL2 represses the promoter activity of *StAR*, whereas it directly increases sirtuin 1 (*SIRT1*) transcription (Benayoun et al., 2009; Pisarska, Bae, Klein, & Hsueh, 2004). In order to confirm that mutant proteins alter the expression of target genes, the expression of endogenous *StAR* and *SIRT1* mRNA was measured by Quantitative real‐time PCR (qPCR). Transfected cells were incubated for 36 hr, and then total mRNA was extracted using Trizol (Invitrogen, Carlsbad, CA, USA). RNA (1.5 μg) was reverse transcribed to synthesize cDNA in a 20‐μl reaction mixture. Quantitative SYBR Green real‐time PCR was performed on an Applied Biosystems 7500 system (ABI) using the 20‐μl mixture. The housekeeping gene *GAPDH* was used as a control. The primer sequences were as follows: *StAR*, forward 5′‐ACATGAAAGGACTGAGGCACC‐3′ and reverse 5′‐CTCCTTCTTCCAGCCTTCCTG‐3′; and SIRT1, forward 5′‐AGCTGGGGTTTCTGTTAGCTG‐3′ and reverse 5′‐GACACAGAGATGGCTGGAACT‐3′.

### 2.7 Statistical analysis

Statistical analysis was performed via Student's *t* test and one‐way ANOVA using SPSS software, version 19.0 (SPSS, Chicago, IL, USA). The differences were considered significant at *p*‐values < .05. The results of the real‐time PCR are expressed as bar graphs.

## 3 RESULTS

### 3.1 Sequencing and in silico analysis

The proband from Family F1 (II1) was found to harbor a heterozygous deletion mutation of *FOXL2*, c.462_468del, leading to a frameshift at codon 156. This mutation was predicted to result in the formation of a truncated FOXL2 protein of 267 amino acids (p.Pro156Argfs*113, Figure 1b). The proband from Family F2 (II1) carried a heterozygous insertion mutation (c.988_989insG), which was predicted to result in an extended protein of 532 amino acids (p.Ala330Glyfs*204, Figure 1b). Pedigree analysis revealed that their parents are all free of the mutations, which indicated that the two novel mutations are de novo in the probands.

**Figure 2:**
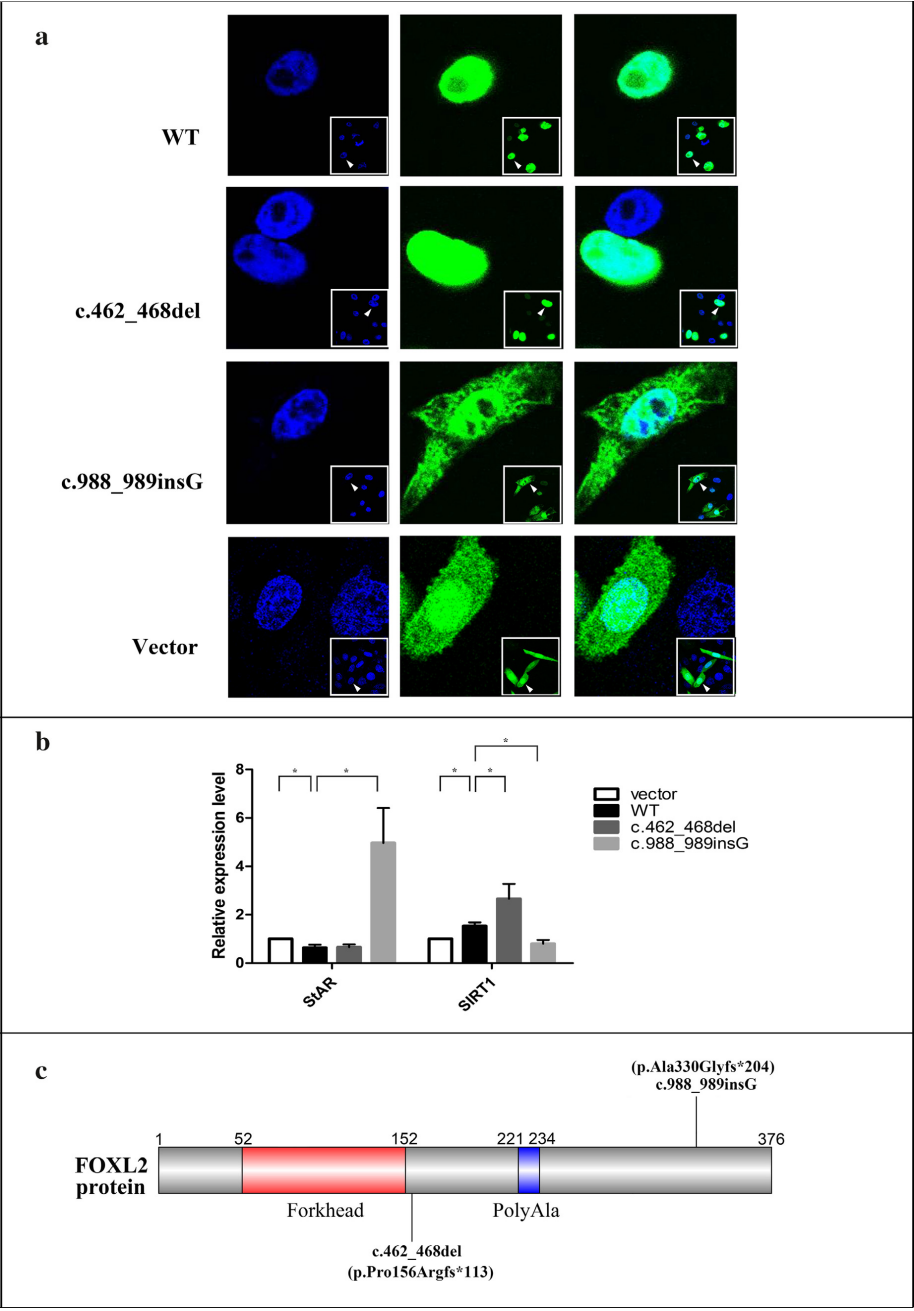
In vitro functional analysis of the two novel mutations and the schematic structure of FOXL2. Panel a: Subcellular localization of wild‐type and mutant FOXL2 proteins. The left panel shows nuclear staining with DAPI. The middle panel corresponds to the subcellular localization of FOXL2 as a fusion protein with GFP. The right panel is a merged image of the previous two images. Panel b: The expression level of endogenous StAR and SIRT mRNA in CHO cells transiently transfected with empty vector, wild‐type FOXL2, and mutant FOXL2 respectively. Statistical significance of the departure of the observed ratio from the expected ratio is represented by *p < .05. Panel c: The schematic structure of *FOXL2* and the location of two mutations identified in this study

The two mutations, c.462_468del and c.988_989insG, were not found in the dbSNP, 1000 Genomes Project, ExAC, and HGMD public databases, suggesting they were novel. All mutations identified in the present study were predicted as pathogenic mutations by bioinformatics analysis. Schematic representation of the *FOXL2* gene and locations of the mutations identified in this study are shown in Figure 2c.

### 3.2 Subcellular location of FOXL2 protein

As expected, the wild‐type FOXL2 protein localizes exclusively in the nucleus in a diffused manner and cells transfected with the empty vector displayed significant cytoplasmic mislocalization. Cells transfected with the mutant construct (FOXL2‐Pro156Argfs‐EGFP) displayed the same pattern as cells transfected with the wild‐type construct, whereas cells transfected with mutant construct containing the FOXL2‐Ala330Glyfs‐EGFP displayed significant cytoplasmic mislocalization as well as nuclear and cytoplasmic aggregation (Figure 2a).

### 3.3 Real‐time qPCR

To assess the effects of the novel *FOXL2* mutations (c.462_468del and c.988_989insG) on transactivation capacity, we performed real‐time qPCR to measure the expression of endogenous *StAR* and *SIRT1* mRNA. When cells were transfected with wild‐type FOXL2, the expression level of endogenous *StAR* mRNA was the lowest, which was consistent with the wild‐type FOXL2 being a repressor of *StAR* expression. The expression level of endogenous *StAR* mRNA in cells transfected with the truncated FOXL2 was similar to that in cells transfected with the wild‐type FOXL2. However, the levels were dramatically elevated in cells transfected with the *FOXL2* mutant encoding the extended protein, suggesting a dominant negative effect over endogenous wild‐type FOXL2 (Figure 2b). The expression level of endogenous *SIRT1* mRNA in cells transfected with the wild‐type FOXL2 was higher than that in cells transfected with the empty vector, which was consistent with the view that *SIRT1* transcription is directly upregulated by the FOXL2 protein. Furthermore, the mRNA expression level of endogenous *SIRT1* in cells transfected with the extended FOXL2‐encoding mutant gene was decreased compared with cells transfected with wild‐type FOXL2. In contrast, the levels were remarkably increased in cells transfected with the truncated FOXL2, suggesting the truncated FOXL2 behaved in a hypermorphic manner on *SIRT1* (Figure 2b).

## 4 DISCUSSION

In the present study, we analysed two Han Chinese families with BPES type I and identified two novel mutations (c.462_468del and c.988_989insG). Immunofluorescence and confocal microscopy revealed that the extended FOXL2, p.Ala330Glyfs*204, induced significant mislocalization and aggregation. Furthermore, we demonstrated that both novel mutations had a strong impact on FOXL2 transactivation abilities. Our data suggest that the novel mutations in *FOXL2* are deleterious and represent disease‐associated mutations in the present probands with BPES and POI.

The forkhead nuclear transcription factor encoded by human *FOXL2* contains two domains: the DNA‐binding forkhead domain (FHD) and the 14‐residue polyAla tract, which are strictly conserved in mammals (Adell & Muller, 2004; Baron et al., 2005; Crisponi et al., 2001;). Investigations of other forkhead transcription factors, such as FOXP2 and FOXP3, have illustrated that the FHD is pivotal for correct nuclear localization (Mizutani et al., 2007; Schubert, Jeffery, Zhang, Ramsdell, & Ziegler, 2001). A frameshift mutation or nonsense mutation is commonly thought to result in truncated or elongated protein and consequent loss of function; different loss‐of‐function proteins may show varying levels of bioactivity (Dipietromaria et al., 2009; Moumné, Fellous, & Veitia, 2005). The novel truncated FOXL2 (p.Pro156Argfs*113) with a complete FHD localizes exclusively in the nucleus in a diffuse manner, which is consistent with its function. The single base insertion (c.988_989insG) is predicted to result in the formation of an elongated FOXL2 protein containing a complete FHD. This phenomenon may induce misfolding that hides the nuclear localization signals or disrupts interactions with nuclear transporters, ultimately resulting in mislocalization, and similar results were observed in our functional study.

Normal bioactivity of FOXL2 is important for ovarian function (Georges et al., 2013). As a nuclear transcription factor, FOXL2 directly represses *StAR* transcription but upregulates *SIRT1* transcription (Benayoun et al., 2009; Pisarska et al., 2004). The *StAR* gene encodes a protein involved in the rate‐limiting step in steroid hormone synthesis that plays an important role in folliculogenesis (Pisarska et al., 2004). *SIRT1* has been found to play a crucial role in the cell stress response, and SIRT1‐knockout mice were found to be infertile (Di Emidio et al., 2014; McBurney et al., 2003). The novel mutations (c.462_468del and c.988_989insG) profoundly impaired the transactivation abilities of FOXL2, which was corroborated by the quantification of endogenous *StAR* and *SIRT1* mRNA levels in CHO cells via qPCR. Therefore, the altered transactivation of *StAR* and *SIRT1* promoters may explain the ovarian phenotype in the present patients. The phenotype of patient from family II was more severe than that of patient from family I, which may be due to a dominant negative effect (Caburet et al., 2001; Méduri et al., 2010). It has reported that a dominant negative effect may be caused by the interaction between mutated FOXL2 protein and the wild‐type protein in the aggregates (Caburet et al., 2001). This effect showed extensive nuclear and cytoplasmic protein aggregation for such a dominant negative effect presented in extended protein and was reported previously (Caburet et al., 2001; Méduri et al., 2010). This is consistent with the view that the mutant FOXL2 proteins implicated in BPES type I strongly decrease the transactivating effect on the promoters of target genes, whereas the mutant FOXL2 proteins implicated in BPES type II are still (at least partially) active (Dipietromaria et al., 2009). Although we did not perform whole‐exome sequencing to eliminate other putative disease‐causative mutations, two mutations identified in this study have been found to have a strong impact on FOXL2 transactivation abilities. Therefore, we suggest that the two mutations of *FOXL2* identified in the present study are pathogenic mutations implicated in BPES type I in the two Han Chinese probands.

Differential diagnosis of BPES types I and II is pivotal for female patients carrying *FOXL2* mutations, as it may allow the patients to predict fertility and plan appropriate therapy. Unfortunately, the genotype‐phenotype correlation between *FOXL2* mutation and the type of BPES developed has not yet been fully delineated. It has been reported that mutations predicted to result in truncated proteins without the FHD are associated with BPES type I, whereas the phenotype associated with mutations leading to truncated or extended proteins with an intact forkhead and poly‐Ala tract were impossible to predict (De Baere et al., 2003). In this study, two novel mutations (c.462_468del and c.988_989insG) resulting in a truncated protein with complete FHD and an extended protein with an intact forkhead and poly‐Ala tract, respectively, were both responsible for BPES type I. Therefore, we demonstrated that a female patient who harbors the novel mutations might have a high risk of infertility, and females with BPES whether onset of POF should be examined by both a clinical geneticist and an endocrinologist for assessment of ovarian function, rather than relying only on molecular testing as a predictor for POI risk.

In conclusion, we identified two novel mutations of *FOXL2* in two Han Chinese families. Our experimental findings suggest that the two novel mutations in the *FOXL2* gene are pathogenic mutations and implicated in BPES type I in the two families, which was confirmed by the in vitro functional analysis. Our findings also contribute to the delineation of genotype‐phenotype correlation between the two novel *FOXL2* mutations and POI, and enrich knowledge of *FOXL2* gene mutation spectrum.

## CONFLICT OF INTEREST

The authors declare no conflicts of interest.
